# COVID-19 and diabetic ketoacidosis: A case series at an urban district hospital in South Africa

**DOI:** 10.4102/safp.v64i1.5590

**Published:** 2022-09-22

**Authors:** Heather N. Dicks, Keshena Naidoo

**Affiliations:** 1 Department of Family Medicine, Northdale Hospital, Pietermaritzburg, South Africa; 2 Department of Family Medicine, College of Health Sciences, University of KwaZulu-Natal, Durban, South Africa

**Keywords:** COVID-19, diabetic ketoacidosis, diabetes, district hospital, HbA1c

## Abstract

**Background:**

Coronavirus disease 2019 (COVID-19) is associated with an increased prevalence and mortality from diabetic ketoacidosis (DKA) globally. With limited access to specialised care, most patients with DKA in South Africa are managed at district hospital level. This study describes the profile of patients admitted to a district hospital in South Africa with DKA and COVID-19 and examines associated risk factors encountered.

**Methods:**

This was a case series of all patients presenting to a district hospital with DKA and COVID-19 infection between July 2020 and July 2021. Data extracted included patients’ demographic profiles, biochemical results, comorbidities and clinical outcomes.

**Results:**

The median age of the 10 patients admitted during the study period was 39 years old (±12), six of whom were male. The hemoglobin A1c (HbA1c) values on admission ranged from 9.7 to 13.8. Five of the patients had pre-existing type 2 diabetes mellitus (DM). Four of the known DM patients were on metformin only, and one was on biphasic insulin. Three patients had other pre-existing comorbidities, two patients with hypertension and one with human immunodeficiency virus (HIV). Three patients demised, two of whom were hypoxic on admission.

**Conclusion:**

Diabetic ketoacidosis appears more commonly in COVID-19 infected patients with type 2 DM and at a young age. Suboptimal glycaemic control was associated with DKA, and hypoxia was a strong predictor for mortality. Treatment inertia was evident in the known DM group, who were on monotherapy despite persistent hyperglycaemia. Greater vigilance is required to detect ketosis in type 2 DM and intensify therapy to improve glycaemic control.

## Introduction

Since the emergence of the novel severe acute respiratory syndrome coronavirus 2 (SARS-CoV-2) in December 2019, it has been evident that diabetes mellitus (DM) increases the risk of severe coronavirus disease 2019 (COVID-19) illness and hospitalisation.^1,2^ Furthermore, SARS-CoV-2 infection may precipitate diabetic ketoacidosis (DKA) in both diabetic and previously undiagnosed diabetic patients with a high risk of fatality.^3,4,5^ Diabetic ketoacidosis is characterised by the triad of hyperglycaemia (glucose > 13.8 mmol/L), ketonaemia or ketosis and a high anion gap metabolic acidosis (HCO3 < 15 mmol/L and/or pH < 7.3).^6,7^

During the pandemic period, the prevalence of DKA almost quadrupled compared to the prepandemic period in hospitalised patients, and the mortality from DKA nearly doubled in patients with COVID-19.^8^ While DKA is typically seen in individuals with type 1 DM, studies report that DKA is more common among COVID-19 patients with type 2 DM.^9,10,11,12,13^ A possible hypothesis for this phenomenon is that SARS-CoV-2 infection might trigger new pathophysiological diabetes-related mechanisms.^5^

There are different speculations regarding COVID-19’s role in triggering DKA. Studies suggest that COVID-19 aggravates beta cell function to precipitate DKA in both pre-existing and newly diagnosed diabetic patients.^14,15^ This is possibly because of the high expression of angiotensin-converting-enzyme 2 (ACE2), the enzyme that is the entry point of SARS-CoV-2, in the pancreas, causing direct injury to the cells. Once the viral complex has entered the cell, ACE2 is downregulated; this leads to unopposed angiotensin II, contributing to impaired insulin secretion and therefore hyperglycaemia.^14,15^

Even though there are case reports of known and previously undiagnosed diabetic patients with COVID-19 presenting with DKA, there is a paucity of data from sub-Saharan Africa on the associated risk factors for DKA in COVID-19 patients.^9^ Some studies in North America indicate that this cohort of patients are young (median age of 47 years) with long-standing hyperglycaemia (median hemoglobin A1c [HbA1c] of 13.8%) on presentation.^10,16^ A recent case series in South Africa reported significantly elevated HbA1c levels (10.9% – 13.4%) among four patients at presentation, of which only one was known to have pre-existing DM.^9^ It is unclear whether COVID-19 unmasks DM or triggers stress hyperglycaemia, a transient state of hyperglycaemia that occurs during serious medical illnesses.^17^

Given the increased incidence and mortality of DKA in COVID-19 patients, it is imperative to investigate the risk factors associated with admission for DKA in order to facilitate early diagnosis and improve clinical outcomes. In this study, a series of 10 patients are discussed, all of whom were admitted to a district hospital in South Africa with DKA and COVID-19. The aim of the case series report is to describe the clinical characteristics of the patients and explore possible risk factors and potential interventions to improve outcomes.

## Research methods

### Study design

This study was a retrospective single-centre case series at an urban district hospital in South Africa. All patients admitted with a diagnosis of DKA between July 2020 and July 2021 were identified from the hospital admissions register. During the study period, all admissions had nasopharyngeal and oropharyngeal swabs sent for identification of the novel coronavirus responsible for COVID-19 disease. Patients with a positive result on the real-time reverse transcription polymerase chain reaction (RT-PCR) assay were then included in the study.

### Setting

The setting was an urban district hospital in South Africa. This busy facility serves a population of approximately 980 000 people and provides general medical, surgical and obstetric services. Approximately 100 patients with DKA are admitted annually. The high incidence of DKA has been attributed to the high prevalence of DM in South Africa.^18^ The hospital has a high care ward but not an intensive care unit (ICU). Patients admitted with DKA are usually managed by the Family Medicine Department in the high care ward.

### Study population and sampling strategy

This case series describes all adult patients (> 18 years old) admitted to the facility with DKA who were also infected with the novel coronavirus. The 12-month study period included the tail end of the first COVID-19 wave, the second wave and the early part of the third COVID-19 wave as experienced in South Africa.^19^ The criteria for diagnosing DKA were as follows:

- random blood glucose > 13.9
- blood pH < 7.3
- ketonuria.

Evidence of coronavirus infection was confirmed by laboratory result (RT-PCR). This test confirms presence of the viral genetic material in the body.^20^ Patients with DKA who were managed as outpatients were excluded from this study. All eligible patients were included in this study.

### Data collection

A data collection tool was used to extract data from selected patient files. Medical records were reviewed, and data were extracted regarding patients’ demographic details, clinical progress and laboratory results.

### Data analysis

Descriptive analysis was conducted on the data. Descriptive statistics were calculated for all variables, with mean ± standard deviation (s.d.) or median and interquartile range reported for continuous variables, and number and percentage of total were reported for categorical variables.

### Ethical considerations

Consent was obtained from the hospital management and the University of KwaZulu-Natal Biomedical Research Ethics Committee (ref. no. BREC/00000907/2019).

## Results

### Patient characteristics

A total of 106 patients were admitted with DKA at the district hospital during the study period (01 July 2020 – 31 July 2021). Ten of the identified patients with DKA also tested positive for the novel coronavirus (9.4%). Table 1 displays the clinical and demographic characteristics of the patient cohort.

**TABLE 1:** Demographic parameters and clinical profiles of patients with COVID-19 and diabetic ketoacidosis.

Parameters measured	*N*	%
**Gender**		
Male	6	60
Female	4	40
**Age**		
< 25	-	0
25–35	3	30
35–45	3	30
> 45	4	40
**Comorbidities**		
HT	2	20
T2DM	5	50
Obesity (BMI > 30 kg/m^2^)	2	20
CKD	nil	nil
Other	1†	10
**Medication**		
Metformin	4	40
Metformin + insulin	nil	nil
Biphasic insulin	1	10
Basal bolus	nil	nil
**Ethnicity**		
African	9	90
Caucasian	1	10
**Mortality**		
Yes	3	30
No	7	70

CKD, chronic kidney disease; HT, hypertension; BMI, body mass index; T2DM, type 2 diabetes mellitus; HIV, human immunodeficiency virus.

† , HIV.

As indicated in Table 1, six of the patients (60%) were under 45 years old. There were six male patients and four female patients. Nine of the patients were documented as African and one Caucasian.

Half of the patients (*n* = 5) were reported to have pre-existing type 2 diabetes mellitus (T2DM), for which they were on treatment. One of the known T2DM patients was on biphasic insulin only, and the other four were on metformin. None of the known DM patients were on two or more antidiabetic agents. Two of the patients with DM had pre-existing hypertension, and one DM patient was also human immunodeficiency virus (HIV)-positive. All of the patients with comorbid conditions were on treatment prior to admission.

The five patients with no previous history of DM reported no symptoms or signs suggestive of diabetes mellitus prior to their admission, nor did they have any other chronic conditions. Only one of the previously undiagnosed diabetic patients was noted to be obese, and two in the known diabetic group were obese. No other anthropometric measurements were recorded.

### Clinical features and outcomes

The biochemical markers and clinical outcomes for these patients are tabulated in Table 2. The investigations recorded include the HbA1c, the arterial blood gas results, CRP (C-reactive protein), urinalysis and the capillary blood glucose (glucometer scale 2 mmol/L – 30 mmol/L) on admission.

**TABLE 2:** Biochemical markers on presentation and clinical outcome.

Patient ID	Age	Clinical presentation	Pre-existing conditions	HbA1c %	CRP	pH	Bic	AG	Capillary glucose	Ketonuria	Sats % R/A	GCS	LOS	Outcome
1	28	Fatigue, sore throat	nil	12.6	342	7.00	-	18	29.6	2+	96.0	15.0	10.0	DC home
2	59	Confusion, cough	nil	13.8	244	7.23	15.0	33	HI	3+	68.0	12.0	1.0	Death
3	61	Pain, vomiting and diarrhoea	nil	13.2	160	6.98	9.0	35	27.0	1+	96.0	15.0	3.0	TF field
4	57	Fatigue	T2DM, HIV	11.9	351	6.87	6.0	33	27	3+	100.0	14.0	5.0	TF field
5	49	SOB, body ache	T2DM, HT	11.9	77	7.29	19.0	20	19.1	1+	96.0	15.0	2.0	TF field
6	36	SOB, cough	T2DM	11.3	307	6.87	6.0	46	HI	2+	96.0	15.0	1.0	TF ICU
7	31	SOB cramps	Nil	12.3	-	6.99	6.0	31	23.8	3+	94.0	15.0	3.0	DC home
8	28	Vomiting, body ache	nil	11.1	44	7.07	8.0	43	31.1	2+	99.0	15.0	3.0	DC home
9	39	Vomiting, body ache	T2DM, HT	11.6	3	6.88	6.0	30	HI	3+	100.0	14.0	4.0	Death
10	40	SOB, cough	T2DM	9.7	210	7.00	8.0	28	18.2	2+	53.0	15.0	1.0	Death
Mean	42.8	-	-	11.9	193	7.02	9.2	31	-	-	89.8	14.5	3.3	-
Median	39.5	-	-	11.9	210	6.99	8.0	32	-	-	96.0	15.0	3.0	-

SOB, shortness of breath; LOS, length of stay; TF, transferred to field hospital; DC, discharged home; CRP, C-reactive protein; Bic, bicarbonate; AG, average glucose; GCS, Glasgow Coma Scale; HT, hypertension; ICU, intensive care unit; T2DM, type 2 diabetes mellitus.

All of the patients were determined to have DKA as evidenced by the presence of hyperglycaemia, ketonuria and acidosis on arterial blood gas specimens. One of the patients had no documented bicarbonate levels on admission but was diagnosed with DKA on clinical features. The average pH on admission was 7.02 (range from 6.87 to 7.29). The patients with the two lowest pH values on presentation (pH of 6.88 and 7.00) both demised. The third patient who demised had an initial pH of 7.23.

The clinical presentation of patients: six of the patients presented with symptoms suggestive of COVID-19 infection (i.e. sore through, cough and shortness of breath). Hypoxia, as denoted by oxygen saturation of less than 90% on room air, was only present in two of the patients, both of whom demised.

Despite seven patients requiring step-up care in an ICU, as indicated by national guidelines, only one was successfully transferred to ICU, where he recovered in seven days and was discharged.^21^ Although it is a recommendation for patients with severe DKA to be managed in an ICU setting, only three of the non-COVID-19 DKA patients were transferred to ICU during the study period.^22^ Under normal circumstances, patients with DKA are managed in the high care ward at the district hospital. Only 1 of the 10 patients in this case series was managed in the high care ward; the rest were managed in the COVID-19 isolation wards. Three of the patients recovered in the district hospital ward and were discharged. Three of the patients were transferred to the field hospital from the district hospital, where management was continued. Three patients demised at the district hospital.

Two of the three patients who demised had severe DKA; the third patient who demised had features of combined DKA/hyperosmolar hyperglycaemic state (HHS).

The average length of stay in hospital was 3.3 days, ranging from 1 to 10 days. The short duration of hospital stay was highly influenced by the COVID-19 pandemic. The district hospital provided acute care and thereafter patients with COVID-19 were stabilised and transferred to one of the COVID-19 field hospitals.

Of note, all of the patients (both with known and previously undiagnosed DM) were noted to have abnormal HbA1c values on admission ranging from 9.7 to 13.8. Also, the known diabetic group had an average HbA1c of 11% and unknown diabetics HbA1c 12.5%, yet the previously undiagnosed diabetic patients had better outcomes (20% mortality) compared to the group of known diabetic patients (40% mortality). This could possibly be because of the relatively short duration of hyperglycaemia in the previously undiagnosed patients, and possible presence of comorbidities and diabetic complications in the known diabetic patients may have adversely affected their prognosis.

## Discussion

This case series provides data on patients admitted to an urban district hospital in South Africa with DKA and COVID-19. Recent systemic reviews concluded that the mortality rate among patients with DKA and COVID-19 was as high as 50%.^11,13^ Furthermore, patients with COVID-19 and DKA have shown more severe complications and have had higher mortality than those without COVID-19.^23,24^ However, there was little representation of the African region in those reviews.^11,13^ The high mortality in our cohort (30%) also suggests that COVID-19 related DKA has a poor prognosis. However, other factors such as the absence of ICU facilities and suboptimal glycaemic control during the pandemic could have also contributed to the poor outcomes.

An unusual finding in this case series is that all the patients had T2DM, half of whom were diagnosed on admission. The majority of patients admitted with DKA elsewhere in South Africa are reported to be type 1 DM patients.^12^ Misra et al. noted an increased incidence of DKA admissions in England during the COVID-19 pandemic, with a higher occurrence among type 2 diabetes and newly diagnosed diabetes than in type 1 diabetes.^25^ However, because of the small sample size in this case series, it is unclear if people with type 2 DM patients infected with COVID-19 in South Africa are more prone to DKA than those with type 1 DM. There is an evident need for improved surveillance and data in the African region, especially at primary care level.

As reported elsewhere, a high HbA1c was associated with risk for hospital admission with DKA and poor outcomes.^10,11,16,23^ Of note, the five patients with known DM had suboptimal glycaemic control, yet 60% were still on oral antidiabetic agents and monotherapy. Further investigation is required into possible physician inertia and lack of adherence to treatment guidelines. It is also possible that other factors during the pandemic contributed to the poor glycaemic control, such as reduced access to chronic care management and more sedentary lifestyles because of social restrictions and lockdown regulations.^23^

In this case series, patients who demised were hypoxic on presentation, or became hypoxic shortly after admission, and those who survived were not oxygen dependent. Studies have shown that the level of hypoxia on presentation to a facility is the most reliable biomarker of critical illness, ICU admission and poor outcomes.^26,27^ Those patients who presented with significant hypoxia requiring more than simple facemask oxygen therapy demised.

It has become evident that type 2 diabetes is one of the leading contributors to poorer outcome in patients with COVID-19. Despite this, severe illness can occur, even in previously healthy individuals, at any age.^28^ Further contributors to severe COVID-19 disease are age, obesity and other comorbidities, with obesity and hyperglycaemia seemingly being independent risk factors for a worsened course of illness.^23^ In this study, comorbidities included hypertension, obesity and HIV. However, the majority of patients were under 45 years old and had no chronic conditions other than DM. Improved glycaemic control alone could potentially result in significantly reduced risk for admission for DKA. There is an evident need for improved attention to community education and health provider adherence to diabetes guidelines.

### Limitations

The sample size in this case series is small, and therefore the characteristics of this cohort of patients is not statistically significant. Furthermore, the patients selected were a convenience sample from one facility and may not be representative of all patients with DKA and COVID-19. The period of sampling occurred during the height of the COVID-19 pandemic and therefore other factors such as staff shortages and changes in facility resources may have influenced the inpatient management and clinical outcomes of the patients.

## Conclusion

This study provides useful insight into the risk factors for admission among patients with both DKA and COVID-19 infection in a sub-Saharan African setting. As there were no type 1 DM patients in this case series, it is unclear how COVID-19 influences DKA in type 1 DM in our setting. A significant finding was the suboptimal glycaemic control in all the known diabetic patients who were on monotherapy despite markedly elevated HbA1c levels. Greater attention is required to intensify treatment for poorly controlled type 2 DM patients at primary care level.
